# Antihyperglycemic Activity of *Alchemilla viridiflora* Herb Methanol Extract in Streptozotocin-Induced Diabetic Male Rats

**DOI:** 10.3390/molecules30132819

**Published:** 2025-06-30

**Authors:** Jelena S. Radović Selgrad, Dušan J. Ušjak, Marina T. Milenković, Neda Lj. Milinković, Radmila M. Janković, Jovan B. Jevtić, Ksenija S. Mileski, Marjan S. Niketić, Tatjana D. Kundaković-Vasović

**Affiliations:** 1 Department of Pharmacognosy, Faculty of Pharmacy, University of Belgrade, Vojvode Stepe 450, 11221 Belgrade, Serbia; jradovic@pharmacy.bg.ac.rs; 2 Department of Microbiology and Immunology, Faculty of Pharmacy, University of Belgrade, Vojvode Stepe 450, 11221 Belgrade, Serbia; dusan.usjak@imgge.bg.ac.rs (D.J.U.); marinama@pharmacy.bg.ac.rs (M.T.M.); 3 Laboratory for Molecular Biology, Institute of Molecular Genetics and Genetic Engineering, Vojvode Stepe 444a, 11042 Belgrade, Serbia; 4 Department of Medical Biochemistry, Faculty of Pharmacy, University of Belgrade, Vojvode Stepe 450, 11221 Belgrade, Serbia; nedan@pharmacy.bg.ac.rs; 5 Institute of Pathology “Prof. dr Đorđe Joannović”, Faculty of Medicine, University of Belgrade, dr Subotića starijeg 1, 11000 Belgrade, Serbia; radmila.jankovic@med.bg.ac.rs (R.M.J.); lordstark90@gmail.com (J.B.J.); 6 Institute of Botany and Botanical Garden “Jevremovac”, Faculty of Biology, University of Belgrade, Takovska 43, 11000 Belgrade, Serbia; ksenija.mileski@bio.bg.ac.rs; 7 Natural History Museum, Njegoševa 51, 11111 Belgrade, Serbia; mniketic@nhmbeo.rs; 8 Department of Chemical and Biological Sciences, Serbian Academy of Sciences and Arts, Kneza Mihaila St. 35, 11000 Belgrade, Serbia

**Keywords:** *Alchemilla viridiflora*, antidiabetic activity, hypolipidemic activity, α-amylase, α-glucosidase, lipase, histological examination

## Abstract

Based on the traditional use of *Alchemilla* L. species for the treatment of diabetes, the effect of the methanol extract of *Alchemilla viridiflora* (AVM) on enzyme activity *in vitro* and its impact on blood glucose levels *in vivo* were investigated. Diabetes was induced in male Sprague-Dawley rats using streptozotocin. AVM was administered to both normal and STZ-diabetic rats for 20 days at three different doses. Blood glucose levels and body weights of the treated animals were monitored throughout the experiment. After 20 days, serum insulin, cholesterol, triglycerides, and high- and low-density lipoproteins were measured. In addition, a histological analysis of the pancreas was performed. The AVM demonstrated inhibitory effects on the activities of all tested enzymes. In the *in vivo* experiment, a statistically significant reduction in body weight was observed in the AVM-treated animals at all three doses compared with the normal control group. Notably, a dose of 200 mg/kg significantly decreased blood glucose levels on both the 10th day and 20th day (*p* < 0.05). However, the extract showed no statistically significant effects on the tested biochemical parameters. Overall, the results of this study suggest that AVM has potential for the treatment of hyperglycemia associated with diabetes and obesity.

## 1. Introduction

Diabetes mellitus (DM) is a complex metabolic disorder characterized by fasting hyperglycemia > 7 mmol/L or an incident hyperglycemia > 11 mmol/L [1]. It is the result of impaired insulin secretion and/or insulin action, leading to disturbances in the metabolism of carbohydrates, fats, and proteins [2]. According to the World Health Organization (WHO), the number of people with DM has increased significantly over the years and is expected to continue to rise [3]. Diabetic dyslipidemia, which affects various lipid parameters such as total cholesterol (CH), total triglycerides (TG), high-density lipoproteins (HDL), and low-density lipoproteins (LDL), is also common in people with diabetes [4].

Despite being effective in the treatment of diabetes, conventional drugs are associated with severe side effects, such as hypoglycemic coma and liver or kidney failure [5]. Therefore, herbal products are increasingly favored for the treatment of diabetes due to their efficacy and better tolerability, easy accessibility, affordability, and lesser side effects compared with conventional drugs, and the amount of scientific research regarding the use of herbal medicine in the treatment of diabetes is continuously increasing [6,7]. Nevertheless, the historical use of herbal medicine in the treatment and prevention diabetes is longer compared with conventional drugs [8]. Phytotherapy also implements the herbal formulations of multiple herbs that can synergistically benefit the treatment of diabetes due to its antioxidant, antidiabetic, and hypolipidemic properties [7]. In traditional medicine, more than 1200 plants have been identified that treat diabetes [9,10]. These plants can regulate blood glucose levels through various mechanisms, including insulin-dependent and non-insulin-dependent pathways, as well as by inhibiting certain enzymes [11,12]. Although a substantial amount of the literature exists, the actual clinical effectiveness of medicinal plants in diabetes management remains debated, highlighting the urgent need for more robust evidence-based data [6]. Nevertheless, there are still a considerable number of plants possessing therapeutic uses in diabetes and supported by clinical data. In a randomized, placebo-controlled, crossover, and single blind study, dried leaves of *Ocimum tenuiflorum* L., Lamiaceae showed a significant decrease in fasting and postprandial blood glucose levels and a mild reduction in total cholesterol levels. Supplementation with *O. tenuiflorum* capsules (250 mg twice daily for 8 weeks) decreased plasma insulin and insulin resistance, caused the normalization of serum lipid profile, and reduced body weight and body mass index [6]. In animal studies, the methanol extract of leaves increased glucose uptake and insulin sensitivity, caused regeneration of pancreatic beta cells, and showed α-amylase and α-glucosidase inhibitory activity [8]. The bulbus of *Allium cepa* L., Amaryllidaceae, commonly known as onion, can exert its antidiabetic activity in various forms (extracts, juice, freeze-dried powder, essential oil). For example, consuming 100 g/day of small slices of onion reduces fasting blood glucose in type 1 and type 2 diabetic patients. Also, 7% freeze-dried onion powder added into the control diet of streptozotocin-induced diabetic rats decreases serum cholesterol, triacylglycerol, and LDL-cholesterol, without alterations in cholesterol and HDL-cholesterol levels [6]. Another commonly used spice, garlic, *Allium sativum* L., Amaryllidaceae, in the form of an ethanolic extract, stimulates the secretion of insulin from pancreatic beta cells, sparing the insulin effect. Furthermore, it causes a significant reduction in serum glucose by increasing glucose utilization in streptozotocin-induced diabetic rats [8]. The seeds of *Trigonella foenum-graecum* L., Fabaceae, fenugreek, in the form of extracts, powder, and gum, is a rich source of fibers, which slow the metabolism of carbohydrates via the inhibition of lipid- and carbohydrate-hydrolyzing enzymes and thus can improve insulin sensitivity resulting in reduced insulin levels and lowered blood glucose [13]. Recent systematic reviews and meta-analysis conclude that the consummation of 5–100 g/day of fenugreek seeds may contribute to the management of diabetes via a better glycemic control in patients with type 2 diabetes mellitus, reducing fasting blood glucose and glycated hemoglobin. Also, the seed powder (5% in the diet for 21 days) decreased the level of lipid peroxidation in alloxan-induced diabetic rats and reduced elevated fasting blood glucose levels [6]. Despite phytotherapy offering a promising adjunctive treatment of diabetes, especially by targeting multiple metabolic pathways, phytotherapy should be used with consideration because of the lack of information available about potential herbal–drug interactions and the quality, purity, and standardization of plant extracts, critical for efficacy and safety.

Various species of the genus *Alchemilla* L., Rosaceae, are also traditionally used in the treatment of diabetes. The most widespread species, *A. vulgaris* L., is used as a whole plant in the form of infusion, while the leaves and flowers of species *A. compactilis* Juz. and *A. speciosa* Buser are used in Turkish folk medicine in decoction, infusion, or fresh forms [14,15]. *Alchemilla* L. species are rich in phenolic compounds such as flavonoids, tannins, and phenolic acids [16]. Besides its traditional uses in diabetes, some *Alchemilla* species have confirmed activity *in vitro* as very good α-amylase and α-glucosidase inhibitors, such as *A. vulgaris* L., *A. barbatiflora* Juz. and *A. pseudocartalinica* Juz. [17,18,19,20,21]. However, previous *in vivo* studies have not confirmed the hypoglycemic effect of any species from genus *Alchemilla* [22,23,24].

The pharmacological potential of *Alchemilla viridiflora* Rothm., a species endemic to the Balkans, has not yet been investigated. Previously, it was confirmed that the methanol extract of the aerial parts of *A. viridiflora* is rich in polyphenols, mainly ellagitannins and flavonoids [25,26].

Considering the promising chemical composition of *A. viridiflora* and the traditional use of species from the genus *Alchemilla* in the treatment of diabetes, it is hypothesized that the methanol extract of *Alchemilla viridiflora* (AVM) rich in bioactive phytochemicals exhibits significant inhibitory activity against α-amylase, α-glucosidase, and pancreatic lipase enzymes, thereby reducing carbohydrate and lipid digestion. Furthermore, administration of the extract to diabetic rats would result in improved glycemic control, demonstrating its potential as a natural hypoglycemic agent. Therefore, the primary objective of this study was to quantify the chemical compound of the AVM and to evaluate its inhibitory effects on key digestive enzymes involved in carbohydrate and lipid metabolism *in vitro*. Additionally, this study aimed to assess the hypoglycemic potential of the extract through *in vivo* experimentation using diabetic rat models.

## 2. Results

### 2.1. Main Compound Isolated from Alchemilla viridiflora Methanol Extract

Using a preparative HPLC technique, 2.6 mg of yellowish powdered compound was isolated. The compound was identified by comparing the experimentally obtained spectral data with values from the literature, which showed good agreement. This compound was identified as quercetin-3,4′-dimethyl ether-7-O-glucuronide. The spectral data of the isolated compound are presented below.

Quercetin-3,4′-dimethyl ether-7-O-glucuronide (Figure S1 in Supplementary Materials). UV λmax 254, 358 nm; (Figure S2 in Supplementary Materials); ESI-MS *m/z* 505 [M-H]- (Figure S3 in Supplementary Materials); ^1^H NMR (400 MHz, MeOD) δ 7.74 (s, 1H, H-2′), 7.67 (d, *J* = 8.2 Hz, 1H, H-6′), 6.94 (d, *J* = 8.1 Hz, 1H, H-5′), 6.81 (s, 1H, H-8), 6.49 (s, 1H, H-6), 5.13 (s, 1H, H-1″), 3.94 (s, 3H, CH3O-C4′), 3.82 (s, 3H, CH3O-C3), 3.65-3.48 (m, 4H, H-2″, H-3″, H-4″, H-5″) (Figure S4 in Supplementary Materials); ^13^C NMR (101 MHz, MeOD) δ 178.8, 163.4, 161.3, 157.1, 156.6, 149.9, 147.6, 138.5, 122.5, 121.3, 115.0, 111.6, 106.3, 100.2, 99.6, 94.5, 79.5, 76.1, 73.1, 71.9, 69.8, 59.2, 55.2 (Figure S5 in Supplementary Materials).

^1^H NMR spectral analysis revealed five signals in the aromatic region at δ 7.74 (s, 1H, H-2′), 7.67 (d, *J* = 8.2 Hz, 1H, H-6′), 6.94 (d, *J* = 8.1 Hz, 1H, H-5′), 6.81 (s, 1H, H-8), and 6.49 (s, 1H, H-6), consistent with the substitution pattern of quercetin derivatives. The anomeric proton of the monosaccharide moiety appeared as a singlet at δ 5.13 (s, 1H, H-1″), while the remaining sugar protons resonated as a multiplet in the δ 3.65–3.48 region. These data are in good agreement with previously reported values for quercetin-7-O-glucuronide [27].

The positions of the additional methoxy groups, observed at δ 3.94 and 3.82, were determined from the HMBC spectrum (Figure S6 in Supplementary Materials). The signal at δ 3.94 showed a correlation with a carbon chemical shift at δ 147.5, which also correlated with H-5′, indicating that this methoxy group is located at C-4′. The signal at δ 3.82 correlated with a carbon chemical shift at δ 138.4, a value consistently assigned to C-3 in quercetin derivatives, confirming that the second methoxy group is located at this position.

### 2.2. Quantitative LC-MS Analyses of Alchemilla viridiflora Methanol Extracts

The identification of polyphenols, ellagitannins, and flavonoid glycosides in analyzed AVM was performed by comparing their retention times and UV and MS spectra to data from the literature, commercially available standards, or isolated compounds and described in detail in our previous work [25]. Quantification of the compounds in the extract was performed by the external standard method, using the peak areas of the SIM chromatograms. The regression equations of the calibration curves, their correlation coefficients (R^2^), concentration ranges, limits of detection (LODs), and quantifications (LOQs) are given in Section 4.4. The quantities of identified compounds in analyzed dry extracts, expressed as mg/g of dry extract, are presented in Table 1.

### 2.3. In Vitro Inhibition of Enzymes

AVM showed a significant concentration-dependent inhibitory effect on both α-amylase and α-glucosidase (IC_50_ = 3.69 ± 0.52 and 2.59 ± 0.47 mg/mL, respectively). The extract was more active against α-glucosidase, as the highest percentage of inhibition, approximately 94%, was achieved at a concentration of 8 mg/mL, while twice the concentration of AVM (16.67 mg/mL) was required for the same effect against α-amylase. The extract showed significantly weaker activity compared with the standard inhibitor, acarbose (anti-glucosidase activity with an IC_50_ = 7.71 ± 1.25; and anti-amylase activity with the IC_50_ = 64.36 ± 3.01 µg/mL). AVM also showed significant concentration-dependent inhibitory activity against porcine pancreatic lipase with an IC_50_ value of 2.08 ± 0.04 mg/mL, which was twice as high as the concentration of the reference drug orlistat (IC_50_ = 1.08 ± 0.06 mg/mL).

### 2.4. Normoglycemic and Oral Glucose Tolerance Test

**Figure 1:**
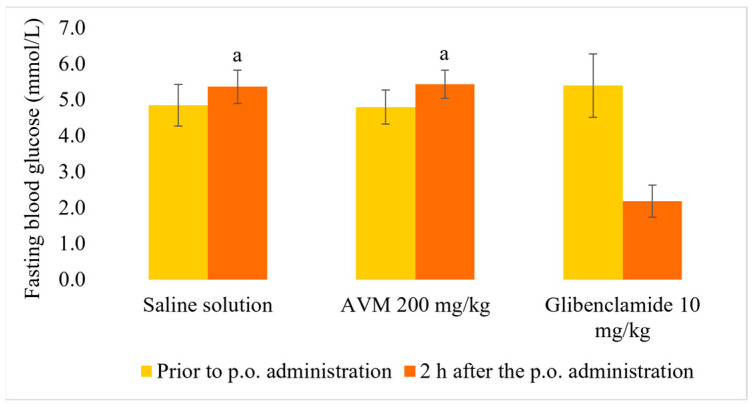
Effect of *Alchemilla viridiflora* methanol extract on fasting glucose levels in normal rats (n = 6). Values are presented as mean ± SEM. One-way ANOVA with Tukey’s post hoc test was used to calculate statistical significance: a *p* < 0.01 compared with group treated with glibenclamide 10 mg/kg b.w.

**Figure 2:**
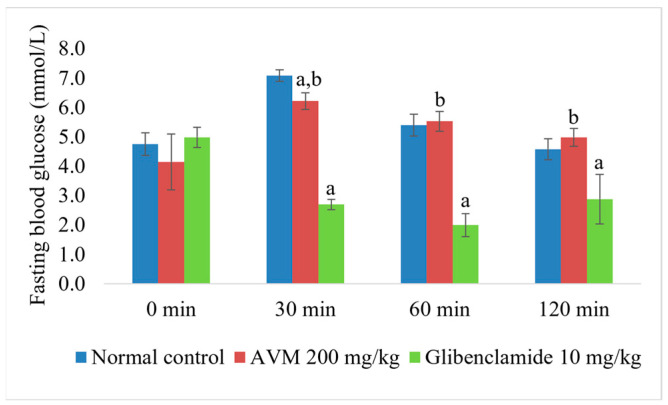
Effect of *Alchemilla viridiflora* methanol extract on oral glucose tolerance test (OGTT) in rats (n = 6). Values are presented as mean ± SEM. One-way ANOVA with Tukey’s post hoc test was used to calculate statistical significance: a *p* < 0.01 compared with normal control group; b *p* < 0.01 compared with group treated with glibenclamide 10 mg/kg b.w.

As shown in Figure 1, treatment with AVM 200 mg/kg b.w. did not result in hypoglycemia in normoglycemic rats two hours after treatment. However, glibenclamide significantly reduced plasma glucose levels compared with the groups treated with both saline and AVM (*p* < 0.01). As presented in Figure 2, thirty min after oral glucose administration, blood glucose levels were significantly lower in rats treated with AVM and glibenclamide than in normoglycemic rats (*p* < 0.01). However, this effect of AVM was not observed 60 and 120 min after glucose administration, in contrast with glibenclamide.

### 2.5. In Vivo Hypoglycemic Effect of AVM

To express the change in values of different treatments, we used delta (Δ) value, expressed as a % and calculated based on the formula:

((T1/T0) − 1) × 100)(1)

where T0 is the average value of the values before the treatment for a given group and T1 is the average value of the variables after the treatment for a given group [28].

Fasting blood glucose levels decreased significantly in rats administered AVM at a dose of 200 mg/kg compared with rats in the diabetic group both on day 10 (Δ = 34.14%, *p* < 0.05) and day 20 (Δ = 44.54%, *p* < 0.05). AVM at a dose of 200 mg/kg showed a similar effect to glibenclamide, which significantly lowered blood glucose levels compared with the diabetic group after day 10 (Δ = 37.41%, *p* < 0.05) and after day 20 (Δ = 47.74%, *p* < 0.05). However, a significant difference between AVM (200 mg/kg) and glibenclamide was not observed on day 10 and day 20 (*p* = 0.999 and *p* = 1.000, respectively). Interestingly, blood glucose levels increased in rats administered AVM at doses of 50 and 100 mg/kg compared with rats in the diabetic group on day 10 of the experiment (Δ = 25.81% and Δ = 26.53%, respectively), but this effect was not statistically significant (*p* = 0.162 and *p* = 0.230, respectively). The blood glucose levels of the rats administered AVM at a dose of 100 mg/kg decreased significantly (Δ = 43.21%, *p* < 0.05) on day 20 compared with the diabetic rats, which is comparable to the effect of AVM at a dose of 200 mg/kg. On day 20, fasting blood glucose levels decreased in all groups of AVM-treated animals, but the differences in effect between all three doses compared with glibenclamide were not statistically significant (*p* = 0.168, *p* = 0.999, and *p* = 1.000, respectively) (Table 2).

The effect size of time is represented by the partial eta squared (η^2^) and values of η^2^ = 0.01, 0.06, and 0.14 represent a small, medium, and large effect size, respectively [29]. Time had a statistically significant large effect on blood glucose levels when AVM and glibenclamide were administered (η^2^ = 0.469, *p* < 0.01). When AVM 200 mg/kg was administered, a statistically significant decrease in blood glucose levels was observed between day 0 and both day 10 and day 20 (*p* < 0.05 each). However, there was no statistically significant difference in blood glucose levels between day 10 and day 20 of the experiment (*p* = 1.00). The same trend was observed with glibenclamide administration (*p* < 0.05 between day 0 and day 10, *p* < 0.01 between day 0 and day 20, and *p* = 1.00 between day 10 and day 20). Interestingly, there was no statistically significant difference in the doses of 100 and 50 mg/kg, where the decrease in blood glucose was observed on day 20, compared with the beginning of the experiment (*p* = 0.411 and *p* = 1.00, respectively).

### 2.6. Effect of AVM on Body Weight

**Figure 3:**
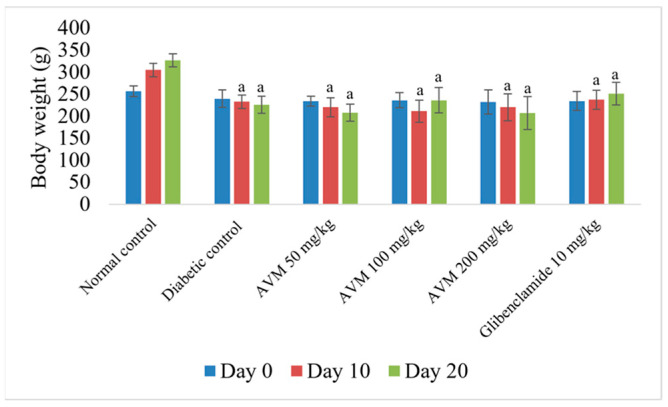
Effect of *Alchemilla viridiflora* methanol extract on body weight in rats (n = 6). Values are presented as mean ± SEM. One-way ANOVA with Tukey’s post hoc test was used to calculate statistical significance: a *p* < 0.01 compared with normal control group.

Regarding the changes in body weight, there was a statistically significant reduction in body weight in the diabetic group and in all experimental groups receiving AVM compared with the normal control on day 10 (Δ = 27.75%, 30.74%, and 27.86%, respectively, *p* < 0.01) and on day 20 (Δ = 36.34%, 27.67%, and 36.60%, respectively, *p* < 0.01). As expected, a slight increase in body weight was observed in the glibenclamide-treated group compared with the rats in the diabetic group at both day 10 (Δ = 1.86%, *p* = 0.999) and day 20 (Δ = 11.26%, *p* = 0.520), but these changes were not statistically significant. The differences between the body weights of rats from the AVM-treated groups (50, 100, and 200 mg/kg b.w.) in relation to glibenclamide on day 20 were also not statistically significant (*p* = 0.053, *p* = 0.909, and *p* = 0.063, respectively) (Figure 3).

### 2.7. Effect of AVM on Biochemical Parameters

**Figure 4:**
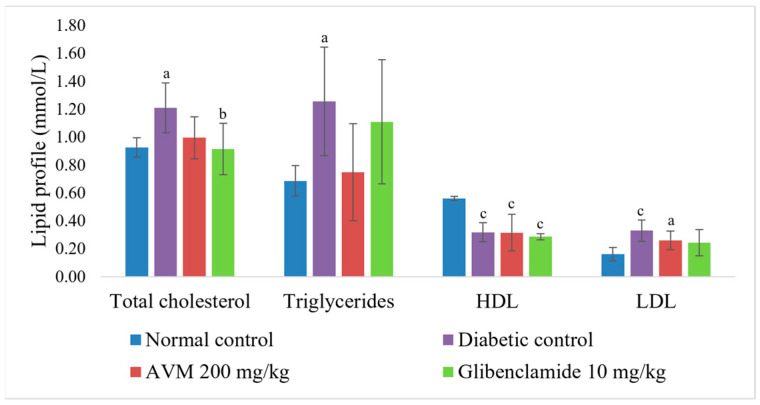
Effect of *Alchemilla viridiflora* methanol extract on lipid status in rats (n = 6). Values are presented as mean ± SEM. One-way ANOVA with Tukey’s post hoc test was used to calculate statistical significance: a *p* < 0.05 compared with normal control group; b *p* <0.05 compared with diabetic control group; c *p* < 0.01 compared with normal control group.

In the groups administered AVM 200 mg/kg (0.6 ± 0.5 pmol/L, *p* < 0.01) and glibenclamide (1.4 ± 1.1 pmol/L, *p* < 0.01), a statistically significant decrease in insulin levels was observed compared with the normal control group (4.7 ± 0.8 pmol/L). A statistically significant increase in insulin levels was only observed in the glibenclamide-treated group compared with the diabetic control group (0.2 ± 0.1 pmol/L, *p* = 0.04). However, a statistically significant difference between AVM 200 mg/kg and glibenclamide was not observed (*p* = 0.259). As shown in Figure 4, there was no statistically significant change in lipid status with the administration of AVM (200 mg/kg) compared with the diabetic group. On the other hand, after 20 days of the experiment, glibenclamide resulted in a statistically significant reduction in only total cholesterol (Δ = 24.29%, *p* < 0.05) compared with the rats in the diabetic control group.

### 2.8. Histopathological Analysis of Pancreatic Tissue

**Figure 5:**
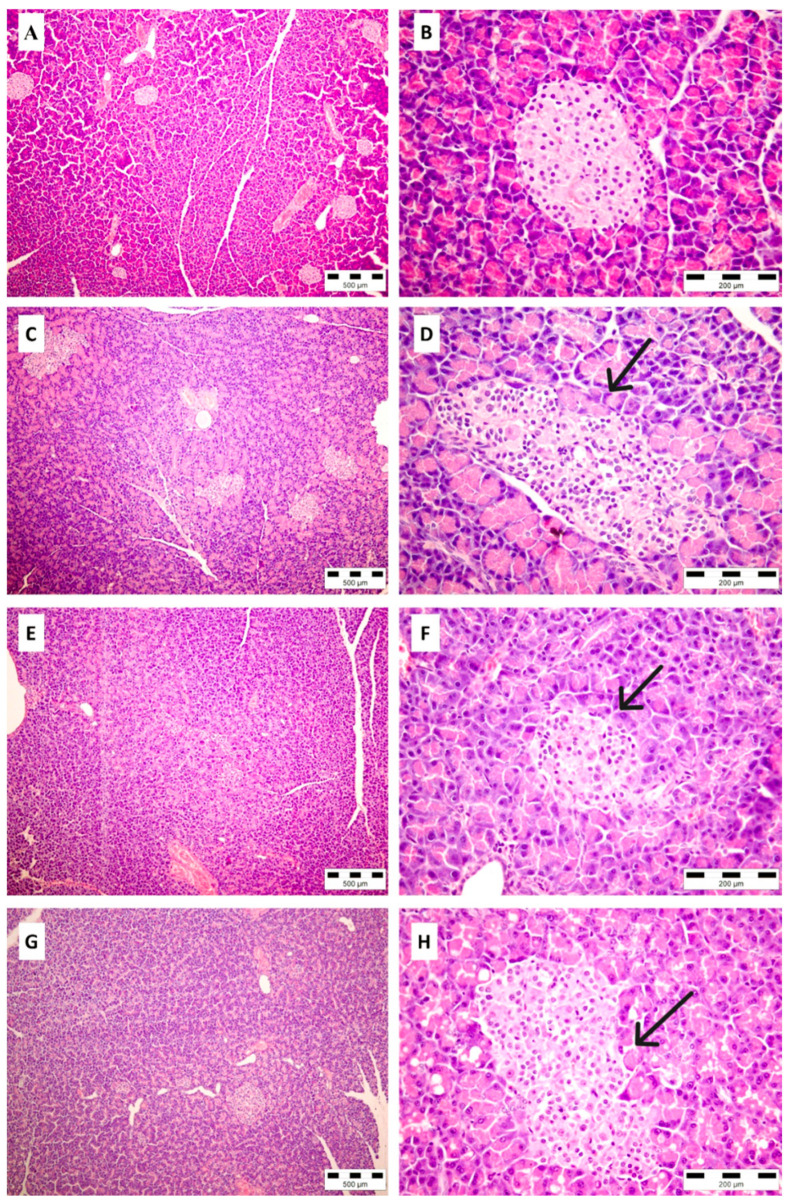
Pancreatic tissue in different experimental groups. Normal control group administered physiological saline solution (**A**,**B**). The diabetic group administered physiological saline solution (**C**,**D**), AVM 200 mg/kg b.w. (**E**,**F**), and glibenclamide 10 mg/kg b.w. (**G**,**H**). ((**A**,**C**,**E**,**G**): ×100, other microphotos: ×400 magnification). The arrows point towards areas of islet cell damage.

After collecting pancreatic tissue samples from normal control, diabetic-, AVM (200 mg/kg)- and glibenclamide-treated groups, histopathological analyses showed normal morphology of Langerhans islets only in the healthy rats (Figure 5A,B). The rats from the diabetic control group showed the most severe changes (Figure 5C,D), including the reduction in a number of pancreatic islets (2.4 ± 0.89), with the collapse of existing islets and an irregular border with surrounded exocrine pancreatic tissue. The vacuolization of endocrine cells was prominent, with evidence of apoptosis in some islets (1.44 ± 0.55). In the groups of diabetic animals treated with AVM (Figure 5E,F) or glibenclamide (Figure 5G,H), histological examination revealed moderate changes in the endocrine pancreas: the number of pancreatic islets was normal or mildly decreased (1 ± 0.71 and 0.5 ± 0.5, respectively). The number of islets in the diabetic group treated with AVM did not differ significantly from the diabetic control group (*p* = 0.061). On the contrary, a significant increase in the number of islets was found in the glibenclamide-treated group (*p* = 0.0178) compared with the diabetic control group. The shape of the islets was often irregular, and the islet’s size was mildly less than normal. Both AVM and glibenclamide significantly reduced islet size and atrophy (1.2 ± 0.45 and 1.17 ± 0.4; *p* = 0.0214 and *p* = 0.0139, respectively) compared with the diabetic control group (2.6 ± 0.05). Vacuolization of islet cells was noted in both the AVM and glibenclamide groups (1.6 ± 0.55 and 2 ± 0.00, respectively) and was less pronounced than in the untreated diabetic group (1.4 ± 0.55), but not statistically significant. However, there was no significant difference between the group treated with AVM or glibenclamide regarding any of the parameters observed.

## 3. Discussion

The effect on the inhibition of the enzymes of carbohydrate metabolism, α-amylase and α-glucosidase, and pancreatic lipase, as well as the hypoglycemic and hypolipidemic effect of the aerial parts of *A. viridiflora*, have not yet been investigated.

According to our previous research, considerable amounts of polyphenolic compounds (233.41 µg gallic acid (GA)/mg dry extract) were detected in AVM, among them tannins (3.74%) and flavonoids (0.30%) [25]. The main class of tannins present in AVM are ellagitannins, of which dimer agrimoniin is prominent as the most abundant (8.43 mg/g of dry extract), accompanied by ellagic acid (4.91 mg/g of dry extract). Agrimoniin, recognized as the chemotaxonomic marker of the Rosaceae family [25], is also found in a similar amount (0.8%) in the methanol extract of the aerial parts of *A. persica* [30]. Among flavonoids, a high amount of miquelianin (3.58 mg/g of dry extract), which is a common compound in many *Alchemilla* species [25], is found in AVM. This amount of miquelianin in AVM is comparable to that measured in both *A. mollis* and *A. persica* aerial parts and methanol/water extracts (0.39 and 0.34 g/100 g, respectively) [31]. Nevertheless, the most abundant flavonoid glycoside in AVM is quercetin-3,4′-dimethyl ether-7-O-glucuronide (8.76 mg/g of dry extract), which is identified and quantified for the first time in the *Alchemilla* species or the Rosaceae family. In addition, brevifolin carboxylic acid, the polyphenol with antiviral and anticancer activity, occurring especially in the species of the families Euphorbiaceae, Rosaceae, and Geraniaceae, was previously only identified in the ethanolic extract of *A. vulgaris* [32,33,34].

Although the AVM inhibited both α-amylase and α-glucosidase in a concentration-dependent manner, the extract showed weaker activity compared with acarbose. The methanol extract of aerial parts of *A. vulgaris* L. showed good inhibition of α-amylase (0.34 mmol acarbose equivalent/g extract) but a weaker effect than the ethyl acetate extract (0.41 mmol acarbose equivalent/g extract), which may be attributed to the presence of a high amount of polyphenols, mainly catechin, and quercetin glycosides [21]. Various extracts from the aerial parts of the species *A. barbatiflora* Juz. also showed significant inhibition of α-glucosidase. The aqueous extract (95.29% and 96.73% at concentrations of 125 and 250 µg/mL, respectively) was more active than the methanol extract (90.56% and 95.36% at the same concentrations, respectively), while the hexane and chloroform extracts showed no inhibition of the enzyme [19].

Regarding pancreatic lipase, AVM showed slightly better activity than the ethanolic extract of *A. vulgaris* (IC_50_ = 3.36 mg/mL), but was weaker than the methanol extract of *A. vulgaris*, which achieved an inhibition of 86.25% at a concentration of 0.5 mg/mL [18,35].

The streptozotocin used in this experiment is a broad-spectrum antitumoral agent that selectively accumulates in pancreatic β-cells, causing their death by various mechanisms, ultimately leading to insulin deficiency and hyperglycemia, but also affects adipose tissue, resulting in significant changes in lipid status [36,37,38,39]. All the above effects of streptozotocin were observed during the experiment, either by severe hyperglycemia and loss of body weight, decreased insulin, and increased CH, TG, and LDL particles in the diabetic control group. The reduction in the number of pancreatic islets observed during histopathological examination of the pancreas also confirms the ischemia caused by streptozotocin.

However, a dose- and time-dependent hypoglycemic effect was observed in AVM, without mortality or other adverse effects during the experiment. Only the highest dose of 200 mg/kg resulted in a statistically significant reduction in fasting blood glucose levels after both day 10 and day 20 compared with the diabetic group. However, the difference in these effects on lowering blood glucose levels is not statistically significant, so it can be concluded that this dose has already achieved the maximum response to hyperglycemia after 10 days. This effect of the extract is comparable to the effect of the reference drug, glibenclamide, which led to the expected statistically significant reduction in glucose levels after the 10th and 20th day of the experiment. However, there was no statistically significant difference between the effect of glibenclamide and AVM after 20 days of the experiment. It is important to keep in mind that the mechanism of action of glibenclamide is not only to reduce hyperglycemia by stimulating insulin secretion but this drug also has hypoglycemia as a side effect [40]. On the other hand, a twice weaker dose, 100 mg/kg, initially led to an increase in glucose levels on day 10 without statistical significance compared with the diabetic group, which could be due to the presence of a lower concentration of active principles (flavonoids and tannins) in the extract [25]. A lower concentration of active principles in the extract had no effect on the reduction of hyperglycemia caused by the progressive deterioration of β-cells. Only after day 20 did this dose lead to a statistically significant reduction in glucose levels compared with the diabetic group. At the end of the experiment, the effect of the 100 mg/kg dose was equivalent to the effect of the 200 mg/kg b.w. dose, meaning that the 100 mg/kg b.w. dose takes twice as long to achieve the same effect. The lowest dose of the extract (50 mg/kg) had no statistically significant effect on the glucose level measured during the experiment.

In the groups administered AVM, a progressive reduction in body weight was observed, despite the hypoglycemic effect. In contrast, an increase in body weight was observed in the group treated with glibenclamide. The potential mechanism of action of the extract of *A. viridiflora* can be assumed by the evidence that the species from the same genus, *A. monticola* Opiz., inhibits the early phase of adipogenesis in adipocytes by suppressing the activity of the (PI3K)/(AKT) metabolic pathway, decreasing the level of intracellular lipids and enhancing lipolysis, leading to a reduction in body weight [41]. As in the species *A. viridiflora*, tannins were the predominant class of secondary metabolites in *A. monticola* (8.5%) [42].

In contrast to glibenclamide, treatment with AVM had no significant effect on insulin levels and lipid status after 20 days of the experiment. The results obtained suggest that the methanol extract of *A. viridiflora* has a similar effect on hyperglycemia as glibenclamide but probably via a different mechanism. This is also supported by the fact that, unlike glibenclamide, the extract does not interfere with normal glucose metabolism, as shown in normoglycemia and oral glucose tolerance test experiments.

This experiment confirmed for the first time the hypoglycemic effect of AVM, but also of other *Alchemilla* species traditionally used to treat diabetes [34]. The study of the blood glucose-lowering effect of the water/methanol extract from the aerial parts of *Alchemilla persica* (100 and 200 mg/kg) on alloxan-induced diabetes (150 mg/kg, i.p.) in Balb/C mice by measuring blood glucose levels before and 1, 2, and 4 h after treatment, an increase in blood glucose levels was also observed 1 h and 2 h after treatment with a dose of 100 mg/kg and 1 h after treatment with a dose of 200 mg/kg, while a slight decrease in blood glucose levels was observed after 4 h with both doses. However, these changes were not statistically significant [22]. The methanol/water extract from the aerial parts of *Alchemilla mollis* (Buser) Rothm. (100 and 200 mg/kg) also had no hypoglycemic effect after 1, 2, and 4 h in Sprague-Dawley rats in which hyperglycemia was induced by alloxan (150 mg/kg, i.p.) [23]. Similarly, the hypoglycemic effect of leaves of the most traditionally used species, *A. vulgaris*, administered as a dietary supplement to rats (6.25% of total body weight) and in the form of decoction (1 g/400 mL water) could not be confirmed after 12 days of treatment in mice with streptozotocin-induced hyperglycemia [24].

The promising hypoglycemic effect of AVM and the inhibition of digestive enzymes can be attributed to the presence of flavonoids (quercetin derivatives) and ellagitannins, identified and quantified in the extract. The flavonoids exert their antidiabetic effects by regulating carbohydrate digestion, insulin signaling and secretion, glucose uptake and improving cell proliferation, promoting insulin secretion, reducing apoptosis, and reducing hyperglycemia by regulating glucose metabolism in the liver. In addition, quercetin and its derivatives have been shown to decrease glucokinase activity and reduce hyperglycemia by stimulating the GLUT 4 transporter, hepatic gluconeogenesis, and glycogenolysis while increasing hepatic glucose uptake in streptozotocin-induced diabetic rats [43].

The two most abundant flavonoid glycosides identified and quantified in AVM are quercetin 3-O-glucuronide, miquelianin, and quercetin-3,4′-dimethyl ether-7-O-glucuronide. According to the literature, in order for quercetin glycosides exert their antidiabetic effects, they need several structural characteristics. The presence of hydroxyl groups at the C3′ and C4′ at B ring and at the C5, C6, and C7 at A ring positions are required for flavonoids to exhibit antidiabetic activity such as against glycogen phosphorylase, aldose reductase, advanced glycation end products (AGE), and α-glucosidase. Also, the presence of the C2–C3 double bond at C ring is crucial for flavonoids’ antidiabetic activity [44], which is observed in the structures of both flavonoids found in AVM. Nevertheless, it was found that glycosylation in both positions C3 (in the case of miquelianin) and C7 (in the case of quercetin-3,4′-dimethyl ether-7-O-glucuronide) can decrease the antidiabetic activity of flavonoids mediated by the inhibition of α-glucosidase [45]. Additionally, glycosylation of the OH group of the C7 position at the A-ring of flavonoids also decreases the inhibition of the formation, accumulation, or action of advanced glycation end products (AGEs), implicated in the development of diabetic complications [46]. However, what is in favor of quercetin-3,4′-dimethyl ether-7-O-glucuronide in AVM is the fact that the methylation of the free hydroxyl groups can prevent the formation of glucuronic acid and sulfate conjugates and thus significantly increase its intestinal absorption and metabolic stability [45]. So, for instance, the methylation of OH groups, particularly in position C3, and at B ring, for instance at C4′, of flavonoids significantly increases the amount of adiponectin released into the medium and the uptake of 2-deoxyglucose into the cells compared with flavonoids having free hydroxy groups that lack these effects. Also, these structural characteristics of flavonoids increase levels of peroxisome proliferator-activated receptor (PPARγ2), a key regulator of insulin sensitivity in adipose tissue, and levels of glucose transporter 4 (GLUT4) and fatty acid-binding protein (aP2), making them promising compounds for the development of antidiabetic agents [47]. Additionally, the introduction of the methoxy group at position C4′ enhances the α-glucosidase inhibitory activity of flavonoids [48].

Similarly, tannins lower glucose levels by delaying intestinal absorption of glucose and have an insulin-like effect on insulin-sensitive tissues. As potent antioxidants, they also prevent the destruction of β cells in the pancreas caused by oxidative stress [49,50]. Tannins, especially hydrolysable tannins, inhibit both α-amylase and α-glucosidase. The mechanism of the inhibitory effect of tannins against these enzymes is based on their ability to precipitate proteins. At low concentrations, the tannins bind to one or more receptor sites on the protein surface, while at high concentrations the tannins form complexes and cross-link with the protein, which in both cases results in the formation of a hydrophobic surface. In this way, they change the conformation of the enzyme and cause aggregation and precipitation, which consequently reduces enzyme activity [51].

Gallic acid and ellagic acid, as well as monomeric ellagitannins, strictinin, and isostrictinin, which each have one hexahydroxydiphenoyl (HHDP) and galloyl moiety in their structure, showed almost no inhibitory effect against α-amylase and α-glucosidase. However, with an increase in galloyl residues, an increase in tannin activity was also observed. Thus, the IC_50_ against α-amylase of the monomers telimagrandin I, ellagic tannin with one HHDP and two galloyl units, and telimagrandin II, with one HHDP and three galloyl units, both identified and quantified in AVM, and rugosin A, with one HHDP and four galloyl units, were 235, 54, and 25 µM. respectively. For α-glucosidase, a similar dependence of activity on concentration can be observed. The IC_50_ values of rugosin D, telimagrandin II, rugosin A, telimagrandin I, and casuarictin were 2, 6, 8, 13, and 22 µM, respectively [52].

It is of particular importance to point out that the dimeric ellagitannin, agrimoniin, which is also the most dominant ellagitannin in the methanol extract of *A. viridiflora*, also showed significant activity in terms of inhibition of α-glucosidase, with an IC_50_ of 21.8 µg/mL, compared with the reference drug, acarbose, whose IC_50_ was even ten times higher (294.4 µg/mL) [53].

The role of tannins in hyperglycemia can be observed using the example of the ethanol extract of the plant *Comarum palsutre* L. (Rosaceae), which has a similar chemical composition to the tested methanol extract of *A. viridiflora*. In rats with streptozotocin-induced diabetes (60 mg/kg), the extract of *C. palustre* (400 mg/kg) before removal of tannins showed a significant reduction in blood glucose levels after 21 days (from over 14 to 6.3 mmol/L). Treatment of diabetic rats with agrimoniin (100 mg/kg) also showed a significant reduction in serum glucose levels after only 14 days (6.2 mmol/L) and after 21 days of the experiment (4.8 mmol/L). On the other hand, administration of the extract after removal of tannins (400 mg/kg b.w.) showed low hypoglycemic activity (blood glucose level of 11 mmol/L), clearly indicating that the presence of tannin contributes to the hypoglycemic effect [53].

The present study demonstrated that the methanol extract of *Alchemilla viridiflora* possesses significant *in vitro* inhibitory activity against key digestive enzymes—α-amylase, α-glucosidase, and pancreatic lipase—implying its potential role in modulating postprandial hyperglycemia and lipid metabolism. Furthermore, the *in vivo* investigation in diabetic rats revealed a promising hypoglycemic effect, supporting the traditional use of the *Alchemilla* species in managing diabetes.

Phytochemical analysis indicates the presence of bioactive constituents likely responsible for these effects; however, further research is essential to isolate and characterize the specific compounds involved. Future studies should focus on elucidating the underlying molecular and cellular mechanisms, including potential modulation of insulin signaling pathways (e.g., PI3K/Akt, AMPK), inflammatory mediators, and oxidative stress-related targets. Additionally, transcriptomic and proteomic profiling may provide deeper insights into the systemic effects of the extract.

Overall, *Alchemilla viridiflora* methanol extract emerges as a promising candidate for the development of adjunct therapies in diabetes management, but comprehensive mechanistic and clinical evaluations are needed to validate its therapeutic potential.

## 4. Materials and Methods

### 4.1. Chemicals

α-Glucosidase (EC 3.2.1.20) Type I, from Saccharomyces cerevisiae; α-amylase (EC 3.2.1.1) Type VI B, from porcine pancreas; lipase (EC 3.1.1.3) Type II, from porcine pancreas, p-nitrophenyl-α-D-glucopyranoside, 3,5-dinitro salicylic acid, soluble potato starch, acarbose, dimethyl sulfoxide, p-nitrophenyl butyrate, Tris-HCl buffer, streptozotocin, LC-MS acetonitrile and formic acid, MeOH-d_4_, and quercetin and miquelianin standard substances were purchased from Sigma Chemical Co., St Louis, MO, USA. Gallic and ellagic acid standard substances were obtained from Carl Roth, Karlsruhe, Germany. Other chemicals of analytical grade were procured from indigenous manufacturers.

### 4.2. Plant Material and Extraction Procedure

The flowering aerial parts, including stems, leaves, and flowers, of *Alchemilla viridiflora* were collected in July 2013 from mountain Suva Planina, Serbia at an altitude of 1750 m. The plant material was identified by Dr. Marjan S. Niketić, curator advisor of the Natural History Museum in Belgrade. The plant material was deposited in a publicly accessible herbarium of the Natural History Museum in Belgrade, Serbia, BEO 101546, under deposition number 20130708/1. The aerial parts of *A. viridiflora,* further used for extraction, are relatively tall, up to 50 cm. The stems are densely covered with erecto-patent hairs. The leaves are suborbicular, densely covered with hairs on both sides, with 9–11 ovate-triangular lobes, and with a serrated margin, with 6–9 large, subacute, and unequal teeth. The petioles are densely covered with hairs, while the flower stalks are usually bare. The flowers are 3–4 mm in size and greenish. The hypanthium is densely covered with hairs [54].

The herb was air-dried at room temperature prior to extraction and then pulverized in a laboratory mill to obtain 320 g of dried herbal material. This herbal material was further used for the successive extraction first with cyclohexane (1:10) for 48 h, then with dichloromethane (1:10) for the next 48 h, and finally with methanol (1:10) for the last 48 h. All extractions were conducted at room temperature in a laboratory shaker (Compact shaker KS 15 A control, Edmund Bühler, Hechingen, Germany). To obtain the methanol extract of *A. viridiflora* aerial parts, which was used for further analysis, methanol was removed under vacuum pressure by a laboratory rotary evaporator with water bath (Rotavapor R II, Buchi Corporation, New Castle, DE, USA), at a temperature of 40 °C, and the extract was weighed (AVM = 80.94 g) and stored at 4 °C [55].

### 4.3. Isolation and Identification of Quercetin-3,4′-Dimethyl Ether-7-O-Glucuronide from Alchemilla viridiflora Methanol Extract

The isolation of compound quercetin-3,4′-dimethyl ether-7-O-glucuronide from the methanol extract of *Alchemilla viridiflora* was performed using preparative high pressure liquid chromatography (HPLC) with MS detection, in a 1260/6130 Agilent LC-MS system, equipped with a Zorbax SB-C 18 column (250 × 9.4 mm; 5 μm, Agilent Technologies, Waldbronn, Germany). The compound was at 25 °C with a binary mobile phase of 0.1% solution of formic acid in water (A) and acetonitrile (B). The gradient program was employed with a flow rate of 3 mL/min: 0–30 min from 10 to 25% B; 30–35 min from 25 to 70% B; 35–40 min return to 10% B, with the total run time of 45 min. The injection volume was 50 μL. Time-based fractionation was performed to obtain 2.6 mg of yellowish powdered compound. The UV and MS spectra of the isolated compound were measured under the same chromatographic conditions. The UV spectra were recorded at 220, 280, 350, and 370 nm wavelengths, and the MS spectra were acquired in the *m*/*z* range of 50–2000 *m*/*z* in negative ion mode with a fragmentor voltage of 100 V. One (1H and 13C at 400 MHz and 101 MHz, respectively)- and two-dimensional (HSQC and HMBC) NMR spectra were recorded on a Bruker (Billerica, MA, USA) Ascend™ 400 spectrometer using deuterated methanol (MeOH-d4) as the solvent. The chemical shifts (δ) were expressed in ppm and the coupling constants (J) in Hz. The identity of the compounds was confirmed by comparing their spectral characteristics with previously published literature data.

### 4.4. LC-MS Analysis of Alchemilla viridiflora Methanol Extract

The quantification of previously identified compounds in methanol extract, mainly ellagitannins and flavonoid glycosides, was performed using the liquid chromatography–mass spectrometry (LC–MS) method. Briefly, 10 mg of *Alchemilla viridiflora* methanol extract (AVM) was dissolved in 1 mL of LC-MS grade methanol, and filtered through a 0.45 μm diameter filter before analysis. The analysis was performed on liquid mass chromatograph Agilent Technologies (Waldbronn, Germany) HPLC 1260 Infinity with an automatic injector, diode array, and single quadrupole mass detectors (SinglequadMSdetector6130). Separation was carried out on a Zorbax SB Aq-C18 column (3.0 × 150 mm; 3.5 m) at 25 °C with a binary mobile phase containing mobile phase A: 0.1% solution of formic acid in water and mobile phase B: acetonitrile. The following gradient program was employed: 0–30 min from 10 to 25% B; 30–35 min from 25 to 70% B; 35–40 min to 10% B. The flow rate was 0.3 mL/min and the injection volume was 3 μL. Detection wavelengths were at 280 and 350 nm and in negative mode in a range of 50–2000 *m/z*. Electrospray ionization under atmospheric pressure was performed under a pressure of 40 psi, temperature of 350 °C, and a nitrogen flow of 10 L/min. The deprotonated molecule signals and fragmented ions were obtained under fragmentation voltages of 100 V and 250 V in full scan. Compounds were identified tentatively by comparison with the literature data, based on the comparison of their ultraviolet (UV) and mass (MS) spectra and to those of commercially available standards or previously isolated compounds, as described in detail in our previous work [25]. External standards, as well as the isolated compound, were used for quantification of the compounds. Qualification of the various compounds was expressed according to the various standards, as given in Table 1. The calibration curves for compounds used for the quantification were, respectively, gallic acid (y = 77,392x + 56.134; R^2^ = 1.0000; concentration range: 0.0025–0.25 mg/mL; LOD = 0.002 mg/mL; LOQ = 0.006 mg/mL); ellagic acid (y = 42,643x − 20.41; R^2^ = 1.0000; concentration range: 0.005–0.250 mg/mL; LOD = 0.003 mg/mL; LOQ = 0.010 mg/mL); miquelianin (y = 39,910x + 38.414; R^2^ = 0.9999; concentration range: 0.01–0.50 mg/mL; LOD = 0.010 mg/mL; LOQ = 0.032 mg/mL); quercetin (y = 5300.3x + 297.01; R^2^ = 0.9961; concentration range: 0.025–1.50 mg/mL; LOD = 0.167 mg/mL; LOQ = 0.505 mg/mL) and quercetin-3,4′-dimethyl ether-7-O-glucuronide (y = 14,413x + 94.544; R^2^ = 1.0000; concentration range: 0.026–1.30 mg/mL; LOD = 0.005 mg/mL; LOQ = 0.016 mg/mL); prepared at 280 nm and 235 nm. All data were obtained in triplicate. The results were expressed as mg/g of dry extract.

### 4.5. α-Amylase Inhibitory Assay

The *in vitro* α-amylase inhibition assay was performed using the 3,5-dinitrosalicylic acid (DNSA) method [56]. Precisely, AVM was dissolved in 10% dimethyl sulfoxide (DMSO) prior to dissolving in phosphate buffer pH 6.9 containing 20 mM Na_2_HPO_4_ and 6.7 mM NaCl to obtain extract concentrations between 16.67 and 0.83 mg/mL. An aliquot of 200 µL porcine pancreatic α-amylase solution (1 IU/mL) was mixed with 200 µL extract and incubated at 37 °C for 15 min. Subsequently, 200 µL of an aqueous 1% (*w/v*) starch solution was added to each tube and incubated at 37 °C for a further 10 min. The reaction was stopped by adding 200 µL of a 1% DNSA solution (30 g sodium potassium tartrate tetrahydrate in 20 mL 2 M NaOH and 1 g 3,5-dinitrosalicylic acid in 50 mL water) and the mixture was boiled in a water bath at 85–90 °C for 15 min, cooled to room temperature, and diluted with 5 mL distilled water. Finally, the absorbance of the mixture was measured at 540 nm. A blank control with 100% enzyme activity was prepared by replacing the plant extract with 200 mL buffer. A positive control sample was prepared with acarbose (83.3–2.60 µg/mL). The inhibitory activity of α-amylase was expressed as a percentage of inhibition and calculated using Equation (2):

% Inhibition = ((Ac − As))/Ac × 100(2)

where Ac is the absorbance of the reaction mixture without samples, and As represents the enzyme activity in the presence of different extract concentrations. The extract concentrations leading to 50% inhibition of enzyme activity (IC_50_, mg/mL) were calculated using the linear regression method taking the dependence of α-amylase inhibition percentage on the extract concentration.

### 4.6. α-Glucosidase Inhibitory Assay

Briefly, α-glucosidase (400 mIU/mL) was dissolved in 0.1 M phosphate buffer (pH 6.7). Then, 50 μL of the solution of AVM in phosphate buffer was added to the microtiter plates, so that the final concentrations in each well were 0.5–8 mg/mL. Then, 50 μL of the enzyme solution was added to each well. After incubation at 37 °C for 15 min, 50 μL of a substrate solution of 1.5 mg/mL PNP-G (p-nitrophenyl-α-D-glucopyranoside) in phosphate buffer was added. The absorbances of A1 were measured at 405. After 15 min incubation, the absorbances of A2 were also measured at 405 nm. Acarbose at concentrations of 20–200 µg/mL was used as positive control. The percentage of enzyme inhibition was calculated according to Equation (3):

% Inhibition = ((A2s − A1s))/((A2b − A1b)) × 100(3)

where A1b, A2b and A1s, A2s represent the absorbances of blanks (phosphate buffer, DMSO, enzyme solution, and substrate PNP-G) and sample, respectively.

IC_50_ values (mg/mL) were calculated by the linear regression method using the dependence of the percentage of inhibition of α-glucosidase to the concentration of the extract [57].

### 4.7. Pancreatic Lipase Inhibitory Assay

The pancreatic lipase inhibitory activity of AVM was determined spectrophotometrically [58,59]. Briefly, AVM (0.25–12.5 mg/mL) and porcine pancreas lipase (2 mg/mL) were dissolved in Tris-HCl buffer (50 mM, pH 7.4–8), while the substrate p-nitrophenyl butyrate (p-NPB, 10 mM) was dissolved in a buffer-DMSO solution (1:1). Then, 100 µL of the dissolved extracts and 25 µL of the enzyme solution were mixed and incubated at 37 °C for 20 min. After the addition of 10 µL of p-NPB, the plates were incubated at 37 °C for 30 min to perform the substrate hydrolysis. The absorbance of the yellow chromophore p-nitrophenol formed was determined at λ = 405 nm. Orlistat was used as a positive control.

Results were expressed as IC_50_ inhibitory concentration (mg/mL) calculated according to the following Equation (4):

% Inhibition = ((Ac))/((As − Ac)) × 100(4)

where Ac is the absorbance of the reaction mixture without samples, and As represents the enzyme activity in the presence of various extract concentrations.

All enzyme inhibitory analyses were performed in triplicate.

### 4.8. Experimental Animals

Sprague-Dawley rats (male, eight weeks old), total of 36, were purchased from the Department of Laboratory and Experimental Animal Breeding, Charles River Laboratories, Calco, Italy, and housed in the animal house at a controlled ambient temperature (25 ± 3 °C), relative humidity of 60 ± 5%, and a light/dark cycle. The animals were fed with food pellets and water ad libitum. The experiment was approved by the Ministry of Agriculture, Forestry and Water Management (Veterinary Administration) of the Republic of Serbia and the Institutional Animal Care and Use Committee of the University of Belgrade-Faculty of Pharmacy (approval number: 323-07-08229/2020-05). All procedures were conducted in accordance with the ethical principles of the Rules for Working with Experimental Animals of the University of Belgrade-Faculty of Pharmacy, the guide for the care and use of laboratory animals of the American National Institute of Health (National institute of health guide for the care and use of laboratory animals, 2011), and Directive 2010/63/EU of the European Parliament and of the Council of Europe.

### 4.9. Normoglycemic Study and Oral Glucose Tolerance Test in Rats

To test the effect of AVM on normoglycemia, a total of 18 rats were used. Prior to the experiment, blood samples were taken by the tail vein puncture and fasting blood glucose levels were estimated in all fasted animals. Then, rats were divided into three groups (n = 6) as follows:

Group 1: rats administered 1 mL of saline solution, per os (p.o.) once;
Group 2: rats administered AVM in dose 200 mg/kg b.w., p.o. once;
Group 3: rats administered glibenclamide, 10 mg/kg b.w., p.o. once.

After 2 h of each treatment, blood samples were taken by the tail vein puncture and fasting blood glucose levels were estimated using the commercial glucometer Accu-chek Active^®^ (Roche, Mannheim, Germany) [60].

To perform oral glucose tolerance test (OGTT), the same 18 rats were later used. Prior to the experiment (0 min), blood samples were taken by the tail vein puncture and fasting blood glucose levels were estimated in all fasted animals. Then, rats were divided into three groups (n = 6), in the same manner as in the normoglycemic study, and glucose (2 g/kg b.w.) was administered p.o. to all animals. Blood samples were taken from the tail vein from all animals 30, 60, and 120 min after glucose administration. Blood glucose levels were determined by using glucometer Accu-chek Active^®^ [61].

### 4.10. Hypoglycemic Effect of AVM in Streptozotocin-Induced DM

To test the hypoglycemic effect of AVM, a total of 36 rats were used, divided into six groups (n = 6) as follows:

Group 1: normal control, rats administered saline solution (1 mL/kg b.w./day);
Group 2: diabetic control, rats administered saline solution (1 mL/kg b.w./day);
Group 3: diabetic rats administered AVM 50 mg/kg b.w./day;
Group 4: diabetic rats administered AVM 100 mg/kg b.w./day;
Group 5: diabetic rats administered AVM 200 mg/kg b.w./day;
Group 6: positive control, rats administered glibenclamide, 10 mg/kg b.w./day.

All the solutions were administered to the rats p.o. once daily for 20 consecutive days.

The experimental model of hyperglycemia was induced in overnight fasting rats in all groups, except in Group 1, by a single intraperitoneal injection (i.p.) of 60 mg/kg b.w. of streptozotocin dissolved in 0.1 M citrate buffer, pH 4.5. Hyperglycemia was confirmed by an elevated blood glucose level measured 72 h and 7 days after the administration of streptozotocin. Rats with permanent blood glucose levels above 11 mmol/L were used for further experiment [62].

### 4.11. Body Weight and Biochemical Estimation

Body weight was measured in all 6 tested groups one day before the induction of diabetes and then 10 and 20 days after the induction of diabetes.

Fasting blood glucose levels were measured in all groups early in the morning, 72 h and 7 days after the induction of diabetes and on days 10 and 20 of the experiment.

After 20 days of the experiment, and 6 h after the last oral administration of saline, AVM, and glibenclamide, the rats were sacrificed under general anesthesia (sodium thiopental, 50 mg/kg b.w.). Blood samples from Groups 1, 2, 5, and 6 were collected by cardiac puncture and centrifuged at 2500 rpm for 10 min and further used for the determination of biochemical parameters. The sacrificed animals were set aside in the prescribed manner until the final upload by the Public Utility Company “Veterina Beograd”, Belgrade, Serbia.

Serum insulin levels were measured using the commercial immunochemical chemiluminescence test (Access Ultrasensitive Insulin, Beckman Coulter Inc., Brea, CA, USA) on a Beckman Coulter Access 2 immunochemistry analyzer. Internal quality control using commercial controls (Biorad, Hercules, CA, USA) was performed with each batch of determinations.

Serum CH, TG, LDL, and HDL levels were measured using commercial biochemical assay (BioSystems, Barcelona, Spain) by spectrophotometric methods on Olympus AU400 automated biochemical analyzer. An internal quality control using commercial controls (Beckman Coulter, Inc., Brea, CA, USA) was performed with each determinations batch.

### 4.12. Histological Examination of the Pancreas

After the sacrifice, the pancreas samples were harvested from 4 groups, normal control, diabetic, group administered AVM (200 mg/kg b.w.), and group administered glibenclamide (10 mg/kg b.w.) for histopathological analysis. Samples were fixed in 4% formaldehyde for 72 h. After the conventional ethanol gradient dehydration, the samples were embedded in paraffin and sectioned (5 µm thick) using a microtome (Leica Reinhart Austria and Leica SM, 2000 R, Nussloch, Germany). The obtained sections were stained with hematoxylin and eosin (H&E, Sigma, St. Louis, MO, USA). The tissue section was analyzed under a light microscope (Olympus BX41, Olympus, Tokyo, Japan). Microphotographs of the pancreatic sections were taken at various magnifications using the Olympus DP70 camera (Olympus, Tokyo, Japan). The entire pancreas of the rat was dissected and histopathologically analyzed. The size and number of Langerhans islets were semi-quantitatively assessed. The number and size of Langerhans islets were graded from 0 to 3. Normal number and size of Langerhans islets were designated as 0. Reduction in the number and size was graded (scored) 1 to 3, where grade 1 indicated mild reduction, and grade 3 indicated severe reduction in both parameters. The results were expressed as the mean value of the score. The analysis was conducted independently by two pathologists.

### 4.13. Statistical Analysis

Data distribution was analyzed using the Shapiro–Wilk test. Values of the continuous variables were expressed as mean ± standard deviation (SD). Statistical evaluation of the data was performed using the statistical software SPSS (version 20.00) by one-factor analysis of variance (ANOVA), followed by post hoc Tukey’s test, except for histopathology data where Mann–Whitney U test was performed. *p*-value of less than 0.05 was considered statistically significant. Additionally, to examine the time effect size on blood glucose levels, partial eta squared (η^2^) was calculated using the general linear model with repeated measurements.

## 5. Conclusions

The tested methanol extract of the aerial parts of *A. viridiflora* showed significant anti-amylase and anti-glucosidase activity *in vitro* and in reducing blood glucose levels and aiding diabetes therapy *in vivo* at the doses of 100 and 200 mg/kg after 10 and 20 days. Flavonoids and tannins were active compounds. These experiments help to clarify the traditional use of *Alchemilla* species in the therapy of diabetes. Exploration of the hypoglycemic activity of the dry methanol extract of the aerial parts of *Alchemilla viridiflora* Rothm., Rosaceae, should be further explored to investigate the potential clinical application, mechanisms of action and effects on lipase activity and body weight reduction.

## Figures and Tables

**Table 1:** Content of identified compounds [17] in methanol extracts of *Alchemilla viridiflora*, expressed as mg/g extract, and the standards they are expressed relative to.

Compound	Content	Expressed As
HHDP-hexoside	0.89 ± 0.02 ^a^	Gallic acid
Pedunculagin isomer 1	1.15 ± 0.02	Gallic acid
Pedunculagin isomer 2	1.19 ± 0.02	Gallic acid
Galloyl-HHDP-glucose	1.79 ± 0.03	Gallic acid
Brevifolin carboxylic acid	8.58 ± 0.05	Ellagic acid
Tellimagrandin 1 isomer 1	0.24 ± 0.02	Gallic acid
Sanguiin H-10 isomer 1	0.83 ± 0.09	Gallic acid
Tellimagrandin 1 isomer 2	0.22 ± 0.03	Gallic acid
Galloyl-bis-HHDP-glucose	3.16 ± 0.01	Gallic acid
Sanguiin H-10 isomer 2	1.06 ± 0.01	Gallic acid
Tellimagrandin 2	1.03 ± 0.01	Gallic acid
Miquelianin	3.58 ± 0.02	Miquelianin
Ellagic acid	4.91 ± 0.01	Ellagic acid
Agrimoniin	8.43 ± 0.02	Gallic acid
Quercetin-3-O-pentoside	0.34 ± 0.14	Quercetin
Quercetin-methyl ether glucuronide	<LOQ	Quercetin
Quercetin-3,4′-dimethyl ether-7-O-glucuronide	8.76 ± 0.05	Isolated compound

^a^ The results represent the average of three determinations ± standard deviation (SD).

**Table 2:** The effect of *Alchemilla viridiflora* methanol extract on fasting blood glucose levels in rats (n = 6).

Group	Fasting Blood Glucose Level (mmol/L)		
	Day		
	0	10	20
Normal control group	4.9 ± 0.6	5.0 ± 0.4	5.9 ± 0.3
Diabetic control group	23.8 ± 6.0 ^a^	25.4 ± 3.2 ^a c^	27.6 ± 4.1 ^a c^
Diabetic + AVM 50 mg/kg	20.7 ± 3.4 ^a^	31.4 ± 4.6 ^a d e^	23.3 ± 8.3 ^a^
Diabetic + AVM 100 mg/kg	21.4 ± 2.5 ^a^	30.9 ± 3.8 ^a d e^	15.7 ± 9.8 ^b^
Diabetic + AVM 200 mg/kg	24.8 ± 4.5 ^a^	16.8 ± 6.7 ^a b^	15.3 ± 3.4 ^b^
Diabetic + glibenclamide 10 mg/kg	26.1 ± 5.2 ^a^	15.9 ± 3.7 ^a b^	14.4 ± 6.2 ^b^

Values are presented as mean ± SEM. One-way ANOVA with Tukey’s post hoc test was used to calculate statistical significance: ^a^ *p* < 0.01 compared with normal control group; ^b^ *p* < 0.05 compared with diabetic control group; ^c^ *p* < 0.05 compared with group treated with glibenclamide 10 mg/kg; ^d^ *p* < 0.01 compared with group treated with glibenclamide 10 mg/kg b.w.; ^e^ *p* < 0.01 compared with group treated with AVM 200 mg/kg b.w.

## Data Availability

Data are contained within the article.

## Supplementary Materials

The following supporting information can be downloaded at: https://www.mdpi.com/article/10.3390/molecules30132819/s1, Figure S1: Structure of quercetin-3,4′-dimethyl ether-7-O-glucuronide; Figure S2. UV spectrum of quercetin-3,4′-dimethyl ether-7-O-glucuronide; Figure S3. MS spectrum of quercetin-3,4′-dimethyl ether-7-O-glucuronide; Figure S4. 1H NMR spectrum of quercetin-3,4′-dimethyl ether-7-O-glucuronide; Figure S5. 13C NMR spectrum of quercetin-3,4′-dimethyl ether-7-O-glucuronide; Figure S6. HMBC spectrum of quercetin-3,4′-dimethyl ether-7-O-glucuronide.

## Abbreviations

The following abbreviations are used in this manuscript:

DM	Diabetes mellitus
AVM	*Alchemilla viridiflora* methanol extract
STZ	Streptozotocin
IC_50_	Concentrations of extract that leads to 50% of inhibition
b.w.	Body weight
WHO	World Health Organization
CH	Cholesterol
TG	Triglycerides
HDL	High-density lipoproteins
LDL	Low-density lipoproteins
LC-MS	Liquid chromatography–mass spectrometry
LOD	Limit of detection
LOQ	Limit of quantification
DNSA	3,5-dinitrosalicylic acid
DMSO	Dimethyl sulfoxide
IU	International unit
PNP-G	p-nitrophenyl-α-D-glucopyranoside
p-NPB	p-nitrophenyl butyrate
OGTT	Oral glucose tolerance test
p.o.	Per os
i.p.	Intraperitoneal
H&E	Hematoxylin and eosin
ANOVA	One-factor analysis of variance
GA	Gallic acid
GLUT4	Glucose transporter type 4
(PI3K)/(AKT)	Phosphoinositide 3-kinase/protein kinase B

## References

[1] Alam S., Hasan M.K., Neaz S., Hussain N., Hossain M.F., Rahman T. (2021). Diabetes mellitus: Insights from epidemiology, biochemistry, risk factors, diagnosis, complications and comprehensive management. Diabetology.

[2] Banday M.Z., Sameer A.S., Nissar S. (2020). Pathophysiology of diabetes: An overview. Avicenna J. Med..

[3] Roglić G. (2016). WHO Global report on diabetes: A summary. Int. J. Noncommun. Dis..

[4] Bhowmik B., Siddiquee T., Mujumder A., Afsana F., Ahmed T., Mdala I.A., Do V. Moreira N.C., Khan A.K.A., Hussain A., Holmboe-Ottesen G. (2018). Serum lipid profile and its association with diabetes and prediabetes in a rural Bangladeshi population. Int. J. Environ. Res. Public. Health.

[5] Yedjou C.G., Grigsby J., Mbemi A., Nelson D., Mildort B., Latinwo L., Tchounwou P.B. (2023). The management of diabetes mellitus using medicinal plants and vitamins. Int. J. Mol. Sci..

[6] Governa P., Baini G., Borgonetti V., Cettolin G., Giachetti D., Magnano A.R., Miraldi E., Biagi M. (2018). Phytotherapy in the management of diabetes: A review. Molecules.

[7] Venkateswaran M.R., Vadivel T.E., Jayabal S., Murugesan S., Rajasekaran S., Periyasamy S. (2021). A review on network pharmacology based phytotherapy in treating diabetes- An environmental perspective. Environ. Res..

[8] Choudhury H., Pandey M., Hua C.K., Mun C.S., Jing J.K., Kong L., Ern L.Y., Ashraf N.A., Kit S.W., Yee T.S. (2017). An update on natural compounds in the remedy of diabetes mellitus: A systematic review. J. Tradit. Complement. Med..

[9] Hsu Y.J., Lee T.H., Chang C.L., Huang Y.T., Yang W.C. (2009). Anti-hyperglycemic effects and mechanism of *Bidens pilosa* water extract. J. Ethnopharmacol..

[10] Guasch-Ferré M., Merino J., Sun Q., Fitó M., Salas-Salvadó J. (2017). Dietary polyphenols, mediterranean diet, prediabetes, and type 2 diabetes: A narrative review of the evidence. Oxid. Med. Cell. Longev..

[11] Sun C., Zhao C., Guven E.C., Paoli P., Simal-Gandara J., Ramkumar K.M., Wang S., Buleu F., Pah A., Turi V. (2020). Dietary polyphenols as antidiabetic agents: Advances and opportunities. Food Front..

[12] Lin D., Xiao M., Zhao J., Li Z., Xing B., Li X., Kong M., Li L., Zhang Q., Liu Y. (2016). An overview of plant phenolic compounds and their importance in human nutrition and management of type 2 diabetes. Molecules.

[13] El-Abhar H.S., Schaalan M.F. (2014). Phytotherapy in diabetes: Review on potential mechanistic perspectives. World J. Diabetes.

[14] Jarić S., Mačukanović-Jocić M., Djurdjević L., Mitrović M., Kostić O., Karadžić B., Pavlović P. (2015). An ethnobotanical survey of traditionally used plants on Suva planina mountain (south-eastern Serbia). J. Ethnopharmacol..

[15] Akbulut S., Bayramoglu M.M. (2014). Reflections of socio-economic and demographic structure of urban and rural on the use of medicinal and aromatic plants: The sample of Trabzon Province. Stud. Ethno-Med..

[16] Kanak S., Krzemińska B., Celiński R., Bakalczuk M., Dos Santos Szewczyk K. (2022). Phenolic composition and antioxidant activity of *Alchemilla* species. Plants.

[17] Tadić V., Krgović N., Žugić A. (2020). Lady’s mantle (*Alchemilla vulgaris* L., Rosaceae): A review of traditional uses, phytochemical profile, and biological properties. Lek. Sirovine.

[18] Samah S., Abdullah K., Ream N. (2018). Phytochemical screening of *Alchemilla vulgaris*, *Sophora japonica*, *Crataegus azarolus*, and their inhibitory activity on lipase and α-amylase. Int. J. Appl. Sci. Res..

[19] Renda G., Ozel A., Barut B., Korkmaz B., Soral M., Kandemir U., Liptaj T. (2018). Bioassay guided isolation of active compounds from *Alchemilla barbatiflora* Juz. Rec. Nat. Prod..

[20] Uzunkaya Ç., Gökkaya İ., Akkaya D., Soral M., Seyhan G., Barut B., Yılmaz M.A., Renda G. (2025). Phytochemical analysis and assessment of antioxidant and enzyme inhibitory activity of *Alchemilla pseudocartalinica* Juz. Chem. Biodivers..

[21] Vlaisavljević S., Jelača S., Zengin G., Mimica-Dukić N., Berežni S., Miljić M., Stevanović Z.D. (2019). *Alchemilla vulgaris* agg. (Lady’s mantle) from central Balkan: Antioxidant, anticancer and enzyme inhibition properties. RSC Adv..

[22] Özbilgin S., Özbek H., Kirmizi N.İ., Öz B.E., Kurtul E., Özrenk B.C., Işcan G.S., Acikara Ö.B. (2019). Evaluation of the antidiabetic activity of *Alchemilla persica* Rothm. in mice with diabetes induced by alloxan. Turk. J. Pharm. Sci..

[23] Ozbek H., Acikara O.B., Keskin I., Kirmizi N.I., Ozbilgin S., Oz B.E., Kurtul E., Ozrenk B.C., Tekin M., Saltan G. (2017). Evaluation of hepatoprotective and antidiabetic activity of *Alchemilla mollis*. Biomed. Pharmacother..

[24] Swanston-Flatt S.K., Day C., Bailey C.J., Flatt P.R. (1990). Traditional plant treatments for diabetes. Studies in normal and streptozotocin diabetic mice. Diabetologia.

[25] Radović J., Suručić R., Niketić M., Kundaković-Vasović T. (2022). *Alchemilla viridiflora* Rothm.: The potent natural inhibitor of angiotensin I-converting enzyme. Mol. Cell. Biochem..

[26] Suručić R., Radović Selgrad J., Kundaković-Vasović T., Lazović B., Travar M., Suručić L., Škrbić R. (2022). In silico and *in vitro* studies of *Alchemilla viridiflora* Rothm—Polyphenols’ potential for inhibition of SARS-CoV-2 internalization. Molecules.

[27] O’Leary K.A., Day A.J., Needs P.W., Sly W.S., O’Brien N.M., Williamson G. (2001). Flavonoid glucuronides are substrates for human liver beta-glucuronidase. FEBS Lett..

[28] Okuka N., Ivanović N.D., Milinković N., Polovina S., Sumarac-Dumanović M., Minić R., Djordjević B., Veličković K. (2025). Probiotic supplementation improves hematological indices and morphology of red blood cells and platelets in obese women: A double-blind, controlled pilot study. Metabolites.

[29] Lakens D. (2013). Calculating and reporting effect sizes to facilitate cumulative science: A practical primer for t-tests and ANOVAs. Front. Psychol..

[30] Afshar F.H., Maggi F., Ferrari S., Peron G., Dall’Acqua S. (2015). Secondary metabolites of *Alchemilla persica* growing in Iran (East Azarbaijan). Nat. Prod. Commun..

[31] Kurtul E., Eryilmaz M., Sarialtin S.Y., Tekin M., Acikara O.B., Coban T. (2022). Bioactivities of *Alchemilla mollis*, *Alchemilla persica* and their active constituents. Brazilian J. Pharm. Sci..

[32] Wolniak M., Tomczyk M., Gudej J., Wawer I. (2006). Structural studies of methyl brevifolincarboxylate in solid state by means of NMR spectroscopy and DFT calculations. J. Mol. Struct..

[33] Tian J., Xie Y., Zhao Y., Li C., Zhao S. (2011). Spectroscopy characterization of the interaction between brevifolin carboxylic acid and bovine serum albumin. Luminescence.

[34] Jelača S., Dajić-Stevanović Z., Vuković N., Kolašinac S., Trendafilova A., Nedialkov P., Stanković M., Tanić N., Tanić N.T., Acović A. (2022). Beyond traditional use of *Alchemilla vulgaris*: Genoprotective and antitumor activity *in vitro*. Molecules.

[35] Jamous R.M., Abu-Zaitoun S.Y., Akkawi R.J., Ali-Shtayeh M.S. (2018). Antiobesity and antioxidant potentials of selected Palestinian medicinal plants. J. Evid.-Based Complement. Altern. Med..

[36] Ventura-Sobrevilla J., Boone-Villa V.D., Aguilar C.N., Román-Ramos R., Vega-Avila E., Campos-Sepúlveda E., Alar-cón-Aguilaret F. (2011). Effect of varying dose and administration of streptozotocin on blood sugar in male CD1 mice. Proc. West. Pharmacol. Soc..

[37] Furman B.L. (2015). Streptozotocin-induced diabetic models in mice and rats. Curr. Protoc. Pharmacol..

[38] Lenzen S. (2008). The mechanisms of alloxan- and streptozotocin-induced diabetes. Diabetologia.

[39] Swami S., Sztalryd C., Kraemer F.B. (1996). Effects of streptozotocin-induced diabetes on low density lipoprotein receptor ex-pression in rat adipose tissue. J. Lipid Res..

[40] Al-Karagholi M.A.M., Sode M., Gozalov A., Ashina M. (2019). The vascular effect of glibenclamide: A systematic review. Cephalalgia Rep..

[41] Mladenova S.G., Vasileva L.V., Savova M.S., Marchev A.S., Tews D., Wabitsch M., Ferrante C., Orlando G., Georgiev M.I. (2021). Anti-adipogenic effect of *Alchemilla monticola* is mediated via PI3K/AKT signaling inhibition in human adipocytes. Front. Pharmacol..

[42] Krivokuća M., Niketić M., Milenković M., Golić N., Masia C., Scaltrito M.M., Sisto F., Kundaković T. (2015). Anti-helicobacter pylori activity of four *Alchemilla* species (Rosaceae). Nat. Prod. Commun..

[43] AL-Ishaq R.K., Abotaleb M., Kubatka P., Kajo K., Büsselberg D. (2019). Flavonoids and their anti-diabetic effects: Cellular mechanisms and effects to improve blood sugar levels. Biomolecules.

[44] Shamsudin N.F., Ahmed Q.U., Mahmood S., Shah S.A.A., Sarian M.N., Khattak M.M.A.K., Khatib A., Sabere A.S.M., Yusoff Y.M., Latip J. (2022). Flavonoids as antidiabetic and anti-inflammatory agents: A review on structural activity relationship-based studies and meta-analysis. Int. J. Mol. Sci..

[45] Xiao J., Kai G., Yamamoto K., Chen X. (2013). Advance in dietary polyphenols as α-glucosidases inhibitors: A review on structure-activity relationship aspect. Crit. Rev. Food Sci. Nutr..

[46] Matsuda H., Wang T., Managi H., Yoshikawa M. (2003). Structural requirements of flavonoids for inhibition of protein glycation and radical scavenging activities. Bioorg. Med. Chem..

[47] Matsuda H., Kogami Y., Nakamura S., Sugiyama T., Ueno T., Yoshikawa M. (2011). Structural requirements of flavonoids for the adipogenesis of 3T3-L1 cells. Bioorg. Med. Chem..

[48] Azuma T., Kayano S., Matsumura Y., Konishi Y., Tanaka Y., Kikuzaki H. (2011). Antimutagenic and α-glucosidase inhibitory effects of constituents from *Kaempferia parviflora*. Food Chem..

[49] Sieniawska E. (2015). Activities of tannins—From in vitro studies to clinical trials. Nat. Prod. Commun..

[50] Kumari M., Jain S. (2012). Tannins: An antinutrient with positive effect to manage diabetes. Res. J. Recent Sci..

[51] Kato C.G., Gonçalves G.A., Peralta R.A., Seixas F.A.V., de Sá-Nakanishi A.B., Bracht L., Comar J.F., Bracht A., Peralta R.M. (2017). Inhibition of α-amylases by condensed and hydrolysable tannins: Focus on kinetics and hypoglycemic actions. Enzyme Res..

[52] Ochir S., Nishizawa M., Park B.J., Ishii K., Kanazawa T., Funaki M., Yamagishi T. (2010). Inhibitory effects of *Rosa gallica* on the digestive enzymes. J. Nat. Med..

[53] Kashchenko N.I., Chirikova N.K., Olennikov D.N. (2017). Agrimoniin, an active ellagitannin from *Comarum palustre* herb with anti-α-glucosidase and antidiabetic potential in streptozotocin-induced diabetic rats. Molecules.

[54] Walters S.M., Tutin T., Heywood V., Burges N., Moore D., Valentine D., Walters S. (1981). *Alchemilla*, L. Flora Europaea.

[55] Radović J., Leković A., Tačić A., Dodevska M., Stanojković T., Marinković T., Jelić Č., Kundaković-Vasović T. (2022). Black trumpet, *Craterellus cornucopioides* (L.) Pers.: Culinary mushroom with angiotensin converting enzyme inhibitory and cytotoxic activity. Pol. J. Food Nutr. Sci..

[56] Wickramaratne M.N., Punchihewa J.C., Wickramaratne D.B.M. (2016). In-vitro alpha amylase inhibitory activity of the leaf extracts of *Adenanthera pavonina*. BMC Complement. Altern. Med..

[57] Grozdanić N., Djuričić I., Kosanić M., Zdunić G., Šavikin K., Etahiri S., Assobhei O., Benba J., Petović S., Matić I.Z. (2020). *Fucus spiralis* extract and fractions: Anticancer and pharmacological potentials. J. BUON.

[58] Qiu X.L., Zhang Q.F. (2019). Chemical profile and pancreatic lipase inhibitory activity of *Sinobambusa tootsik* (Sieb.) Makino leaves. Peer J..

[59] Jaradat N., Zaid A.N., Hussein F., Zaqzouq M., Aljammal H., Ayesh O. (2017). Anti-lipase potential of the organic and aqueous extracts of ten traditional edible and medicinal plants in Palestine; a comparison study with orlistat. Medicines.

[60] Belgacem A., Gdara N.B., Khemiri I., Bitri L. (2019). Exploration of hypoglycemic effect of an extract from leaves of a plant from Tunisian pharmacopeia: *Artemisia campestris* (Asteraceae). Afr. Health Sci..

[61] Husain G.M., Singh P.N., Singh R.K., Kumar V. (2011). Antidiabetic activity of standardized extract of *Quassia amara* in nicotinamide-streptozotocin-induced diabetic rats. Phytother. Res..

[62] Gajdosík A., Gajdosíková A., Stefek M., Navarová J., Hozová R. (1999). Streptozotocin-induced experimental diabetes in male Wistar rats. Gen. Physiol. Biophys..

