# Supplementary material for: Antihyperglycemic Activity of Alchemilla viridiflora Herb Methanol Extract in Streptozotocin-Induced Diabetic Male Rats
Source: Molecules. 2025 Jun 30;30(13):2819. doi: 10.3390/molecules30132819 (PMC12251296; doi:10.3390/molecules30132819)
Supplement: Supplementary file 1 [file molecules-30-02819-s001.zip › molecules-3643159-supplementary.pdf]

Supplementary Materials

# Antihyperglycemic activity of *Alchemilla viridiflora* herb methanol extract in streptozotocin-induced diabetic male rats

Jelena S. Radović Selgrad, Dušan J. Ušjak, Marina T. Milenković, Neda Lj. Milinković, Radmila M. Janković, Jovan B. Jevtić, Ksenija S. Mileski, Marjan S. Niketić, Tatjana D. Kundaković-Vasović

Correspondence: tatjana.kundakovic@pharmacy.bg.ac.rs; Tel.: +381 11 3951-351

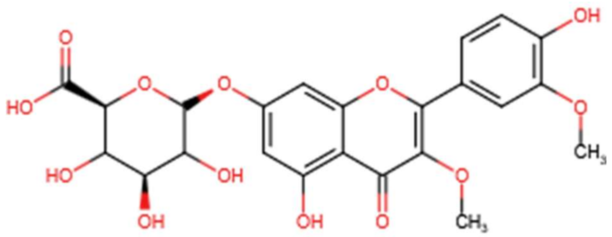


**Figure S1.** Structure of quercetin-3,4'-dimethyl ether-7-O-glucuronide

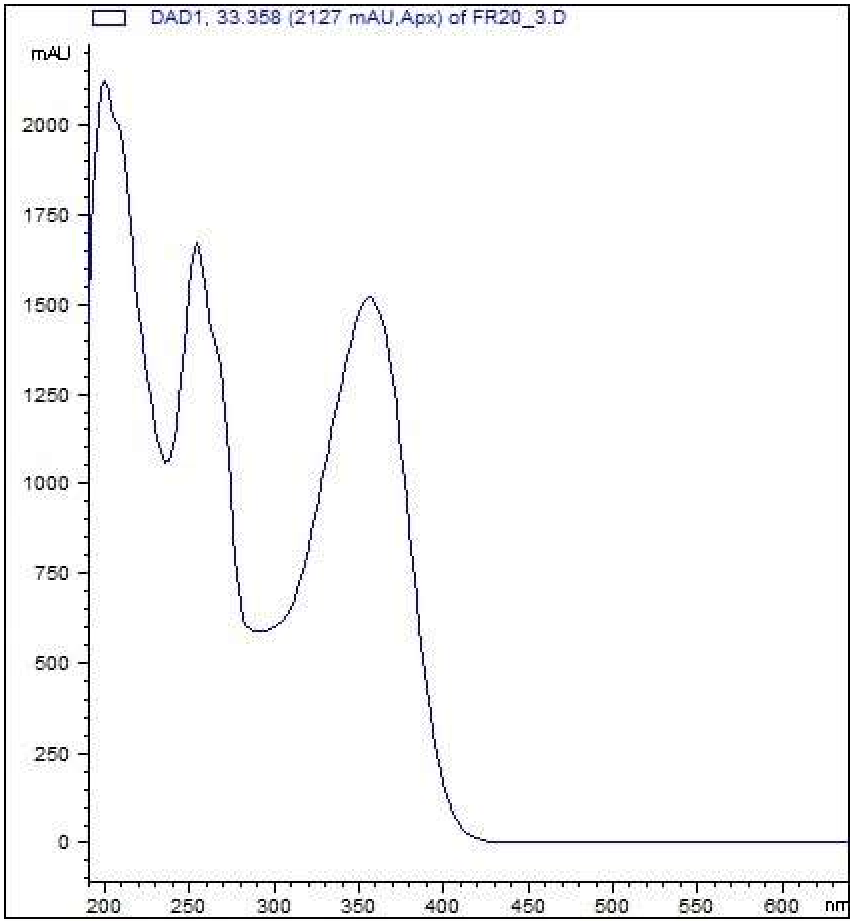

**Figure S2.** UV spectrum of quercetin-3,4'-dimethyl ether-7-O-glucuronide

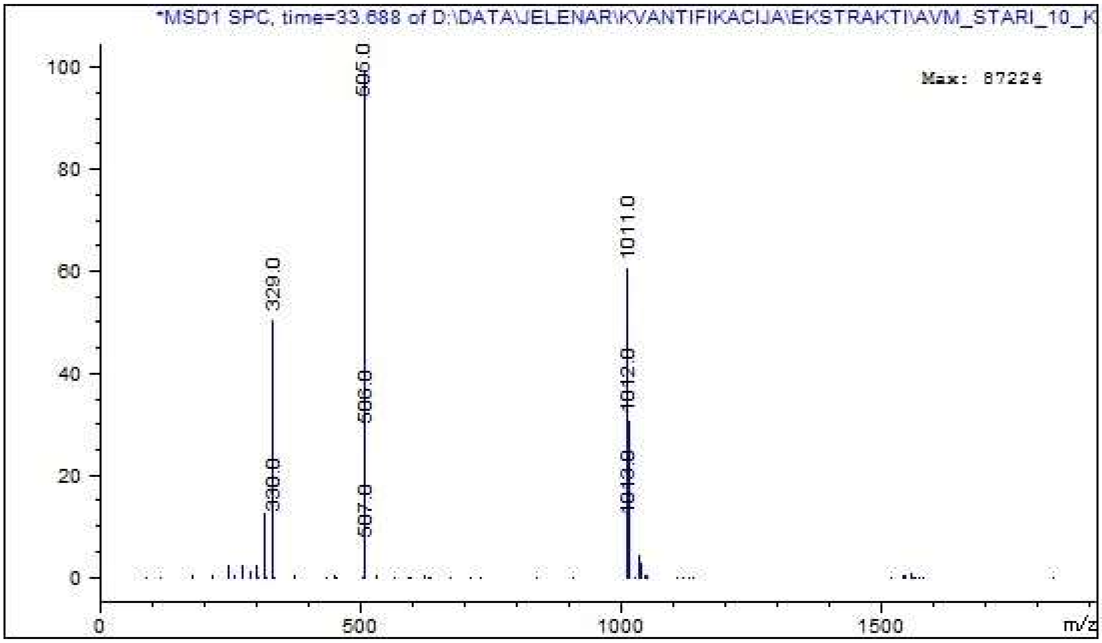


**Figure S3.** MS spectrum of quercetin-3,4'-dimethyl ether-7-O-glucuronide

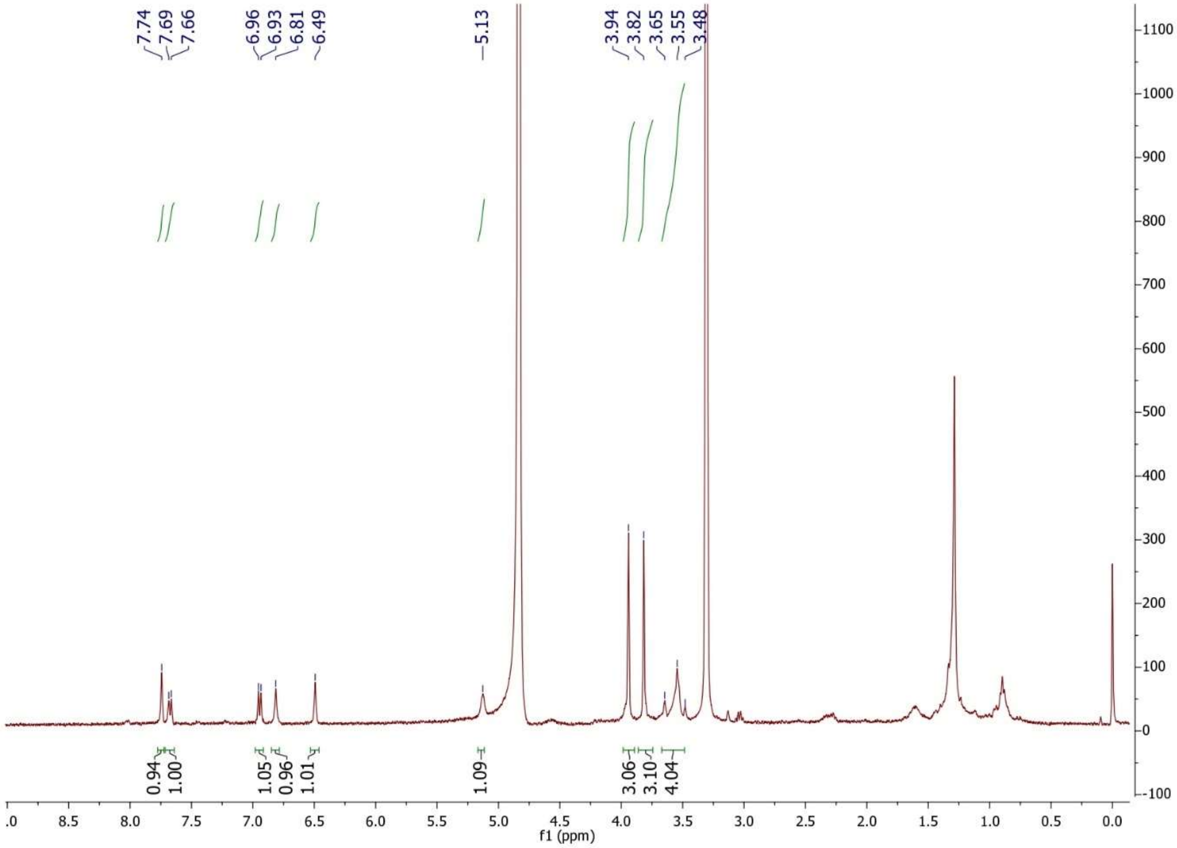


**Figure S4.** $^{1}$H NMR spectrum of quercetin-3,4'-dimethyl ether-7-O-glucuronide

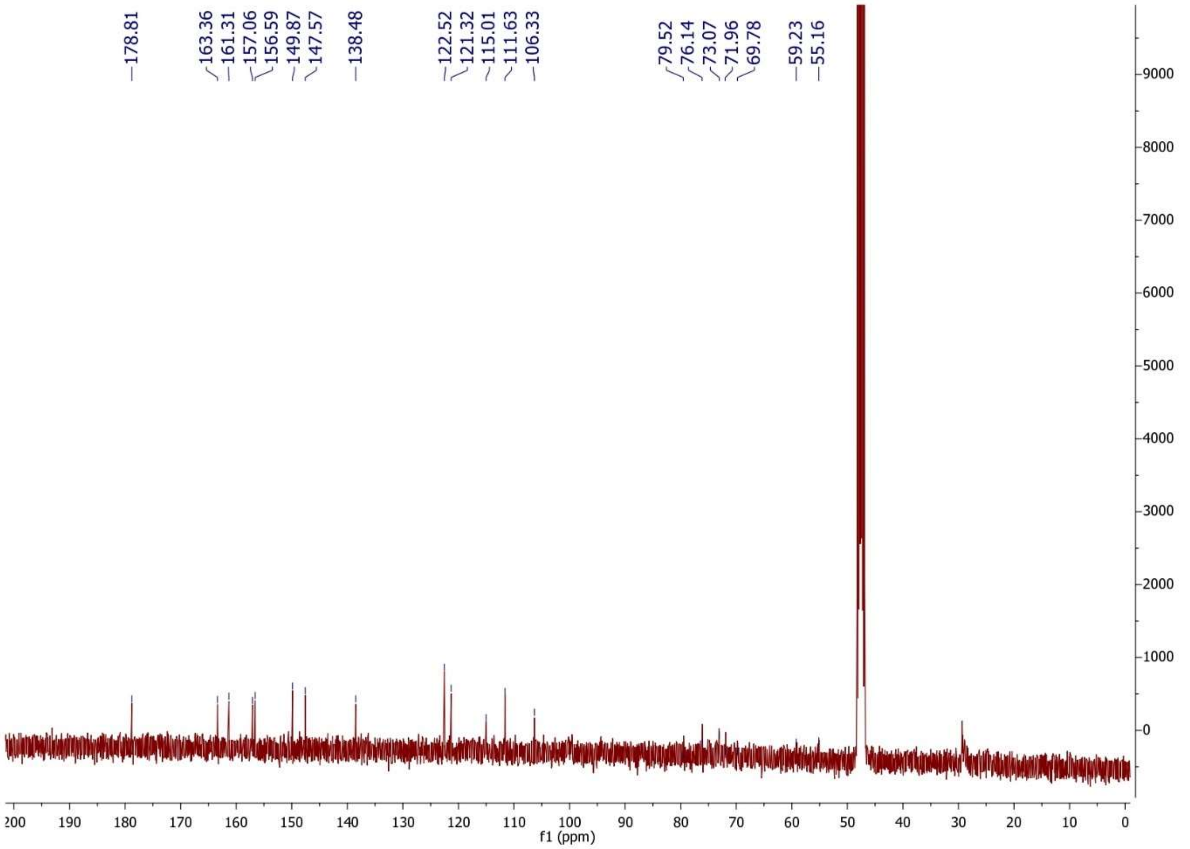


**Figure S5.** $^{13}$C NMR spectrum of quercetin-3,4'-dimethyl ether-7-O-glucuronide

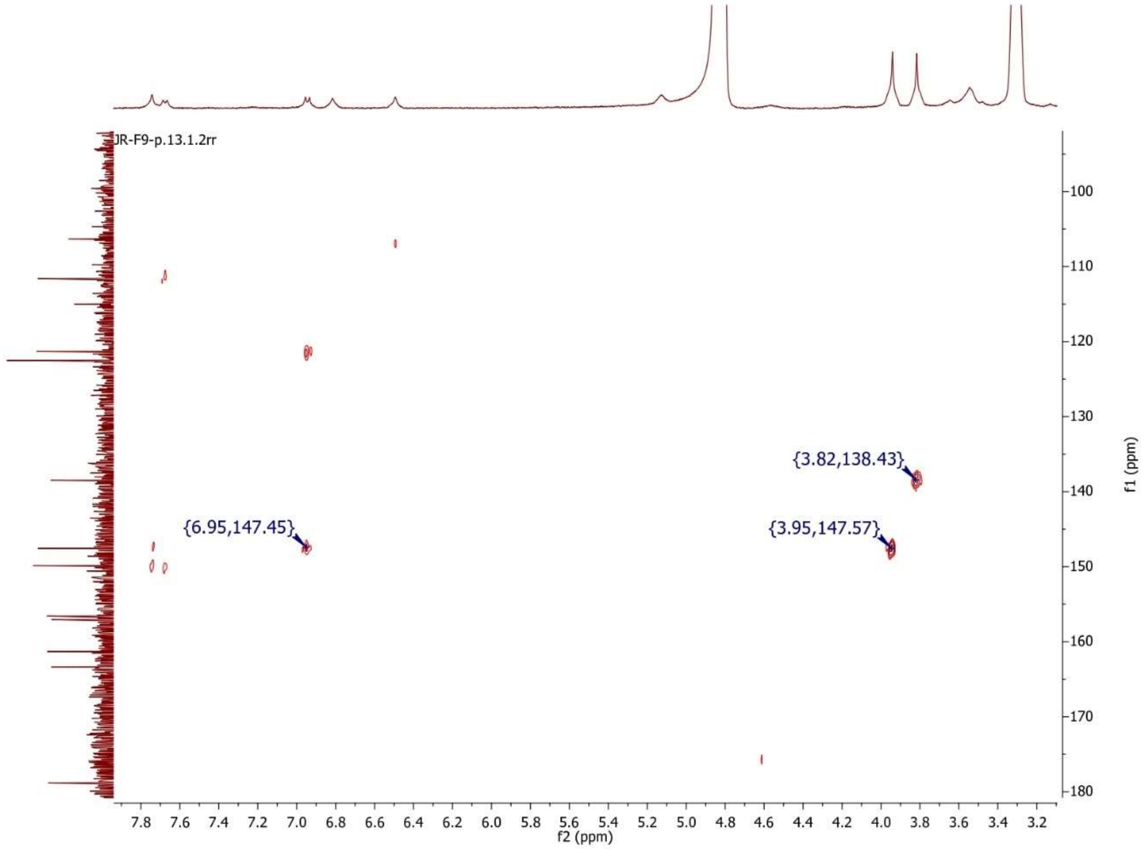


**Figure S6.** HMBC spectrum of quercetin-3,4'-dimethyl ether-7-O-glucuronide
